# Predicting depressive symptom by cardiometabolic indicators in mid-aged and older adults in China: a population-based cross-sectional study

**DOI:** 10.3389/fpsyt.2023.1153316

**Published:** 2023-06-07

**Authors:** Ying Wang, Xiaoyun Zhang, Yuqing Li, Jiaofeng Gui, Yujin Mei, Xue Yang, Haiyang Liu, Lei-lei Guo, Jinlong Li, Yunxiao Lei, Xiaoping Li, Lu Sun, Liu Yang, Ting Yuan, Congzhi Wang, Dongmei Zhang, Jing Li, Mingming Liu, Ying Hua, Lin Zhang

**Affiliations:** ^1^Department of Graduate School, Wannan Medical College, Wuhu, An Hui, China; ^2^Student Health Center, Wannan Medical College, Wuhu, An Hui, China; ^3^Department of Surgical Nursing, School of Nursing, Jinzhou Medical University, Jinzhou, Liaoning, China; ^4^Department of Occupational and Environmental Health, Key Laboratory of Occupational Health and Safety for Coal Industry in Hebei Province, School of Public Health, North China University of Science and Technology, Tangshan, Hebei, China; ^5^Obstetrics and Gynecology Nursing, School of Nursing, Wannan Medical College, Wuhu, An Hui, China; ^6^Department of Emergency and Critical Care Nursing, School of Nursing, Wannan Medical College, Wuhu, An Hui, China; ^7^Department of Internal Medicine Nursing, School of Nursing, Wannan Medical College, Wuhu, An Hui, China; ^8^Department of Pediatric Nursing, School of Nursing, Wannan Medical College, Wuhu, An Hui, China; ^9^Department of Surgical Nursing, School of Nursing, Wannan Medical College, Wuhu, An Hui, China; ^10^Rehabilitation Nursing, School of Nursing, Wanna Medical College, Wuhu, An Hui, China

**Keywords:** depressive symptom, cross-sectional study, cardiometabolic indicators, middle-aged and older adults, obesity

## Abstract

**Objective:**

Depressive symptom is a serious mental illness often accompanied by physical and emotional problems. The prevalence of depressive symptom in older adults has become an increasingly important public health priority. Our study used cardiometabolic indicators to predict depressive symptom in middle-aged and older adults in China.

**Methods:**

The data came from the China Health and Retirement Longitudinal Study 2011 (CHARLS2011), which was a cross-sectional study. The analytic sample included 8,942 participants aged 45 years or above. The study evaluated the relationship between cardiometabolic indicators and depression by measuring 13 indicators, including body mass index (BMI), waist circumference, waist-height ratio (WHtR), conicity index, visceral adiposity index (VAI), Chinese visceral adiposity index (CVAI), lipid accumulation product (LAP), a body shape index (ABSI), body roundness index (BRI), triglyceride glucose index (TyG-index) and its correlation index (TyG-BMI, TyG-waist circumference, TyG-WHtR). Binary logistic regression analysis was used to examine the association between thirteen cardiometabolic indicators and depressive symptom. In addition, the receiver operating characteristic (ROC) curve analysis and area under curve (AUC) were used to evaluate the predictive anthropometric index and to determine the optimum cut-off value.

**Results:**

The study included 8,942 participants, of whom 4,146 (46.37%) and 4,796 (53.63%) were male and female. The prevalence of depressive symptom in mid-aged and older adults in China was 41.12% in males and 55.05% in females. The results revealed that BMI [AUC = 0.440, 95%CI: 0.422–0.457], waist circumference [AUC = 0.443, 95%CI: 0.425–0.460], WHtR [AUC = 0.459, 95%CI: 0.441–0.476], LAP [AUC = 0.455, 95%CI: 0.437–0.472], BRI [AUC = 0.459, 95%CI: 0.441–0.476], CVAI [AUC = 0.449, 95%CI: 0.432–0.467], TyG-BMI [AUC = 0.447, 95%CI: 0.429–0.465], and TyG-waist circumference [AUC =0.452, 95%CI: 0.434–0.470] were weak predictors of depressive symptom (*p* < 0.05) in males. In females, BMI [AUC = 0.470, 95%CI: 0.453–0.486], LAP [AUC = 0.484, 95%CI: 0.467–0.500], TyG-BMI [AUC = 0.470, 95%CI: 0.454–0.487], and TyG-waist circumference [AUC =0.481, 95%CI: 0.465–0.498] were weak predictors of depressive symptom (*p* < 0.05). On the other side, VAI, ABSI, conicity index and TyG index could not predict depressive symptom in middle-aged and older adults.

**Conclusion:**

Most cardiometabolic indicators have important value in predicting depressive symptom. Our results can provide measures for the early identification of depressive symptom in middle-aged and older adults in China to reduce the prevalence of depressive symptom and improve health.

## Introduction

One of the most common mental illnesses worldwide is depression. Depression is generally defined as a mental condition characterized by low mood, decreased interest and impaired cognitive function. Not only does it affect the brain’s perceptual function, but it can also cause mental and physical difficulties in humans. It is also known as depressive symptom or clinical depression (1). A study in 2023 estimated that 3.8% of the world’s population will suffer from depression, including 5.0% of adults and 5.7% of people over 60 (2). The prevalence of depression is related to national cultural differences. The prevalence of depression in older adults is reported to be 11.5% in 2021 in Korea and 23.7% in 2019 in Thailand (3, 4). The prevalence of geriatric depression symptom in China in 2022 is 38.27% (5). Depression will not only impair human function, but also increase the cost of medical, thus bringing a huge burden to both developed and developing countries (6). Researchers conducted a meta-analysis in 2019 that showed life expectancy with disability per 1,000 people with depression and post-traumatic stress disorder was more than five times the current global average burden of disease (7).

Depressive symptom in older adults is related to many factors. Overall, most surveys have shown that basic sociodemographic factors such as gender, age, marriage, education, and occupation were associated with depressive symptom (8–11). For example, one study found that women have a higher rate of depressive symptom than men, possibly because women have higher levels of inflammatory, neurotrophic and serotonergic markers than men (12). In addition, the influence of depressive symptom on metabolic disorders may be more severe in people with a high prevalence of overweight/obesity (13). Multiple studies have shown that waist circumference, elevated triglyceride (TG) levels and fasting blood sugar, as well as the co-existence of obesity and depression, all increase the risk of cardiovascular diseases (14–16). Kinds of literature have described that waist circumference, body mass index (BMI), waist-height ratio (WHtR), lipid accumulation index (LAP) and other indicators have been widely used in epidemiology to measure obesity or central obesity (17–20).

It is important to note that there is no consensus on the relationship between cardiometabolic indicators and depressive symptom. For example, BMI has been utilized in numerous studies looking for a link between obesity and depressive symptom to forecast increased fat buildup. A study in Germany showed that BMI could be used as a predictor of depression (21). In addition, Ma et al. (22) took BMI and WHtR as continuous variables and used logistic regression and RCS models to explore the obvious nonlinear relationship between obesity and depression. In one meta-analysis, the risk of depression rose 38% in both men and women with increased waist circumference (23). Nevertheless, the main limitations of waist circumference include its sensitivity to body size, fat proportion, and distribution, as well as its correlation with BMI (24). In a United States study, Lei et al. (25) found that VAI was associated with the occurrence of clinical depressive symptom after controlling for confounding factors. Previous studies (26, 27) have shown that these cardiometabolic indicators were associated with depressive symptom, in fact, most studies involved only a single or small number of indicators, and there was no consensus on anthropometric indicators that predict depressive symptom (19, 28). In addition, most studies are based on the Western environment, and the results are different due to ethnic and cultural differences. Therefore, in order to promote the management of depressive symptom in middle-aged and older adults, further studies are needed to determine the relevance of cardiometabolic indicators in predicting depressive symptom in middle-aged and older adults.

As far as we know, there is limited research on this aspect in China. Therefore, this study aimed to investigate the association of cardiometabolic indicators (e.g., BMI, LAP, CVAI) with depressive symptoms among middle-aged and older adults in China. To do so, we used a national study examining the health of China’s rapidly aging society. This study can provide a theoretical basis for pre-onset prevention of depressive symptoms and clinical non-drug treatment, enrich the knowledge and understanding of depression-related factors in mid-aged and older adults, make them realize that reducing depressive symptoms can improve the quality of life, and also provide a basis for future clinical intervention and decision-making.

## Methods

### Study design and setting

**Figure 1:**
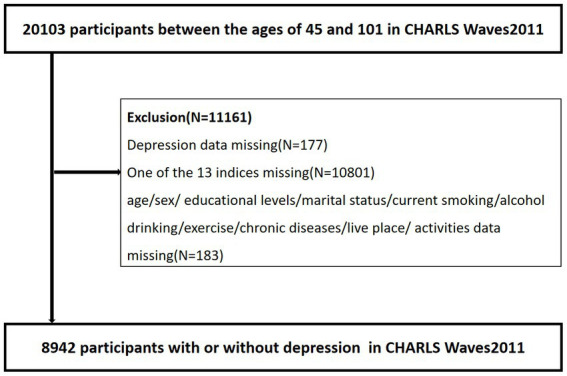
Flow chart of the study participants.

The China Health and Retirement Longitudinal Study (CHARLS) is a nationwide follow-up survey of middle-aged and older adults in China over the age of 45 and their spouses. Every 2 years, the respondents underwent face-to-face Computer Assisted Personal Interviewing (CAPI) were followed up. The baseline survey of CHARLS used a four-stage hierarchical clustering method of random sampling. In the first stage, the regions, rural/urban status, and gross domestic product *per capita* data were used to stratify each county in China. A total of 150 counties were randomly selected as research objects, representing the socio-economic and geographical types of all counties. In the second stage, 3 PSUs were selected for each county-level unit and scale proportional probability sampling was used. In the third stage, the residents in each building were listed, and 24 households were randomly selected from the respondents aged ≥45 years. Finally, a resident aged ≥45 years was randomly selected as the main respondent and his or her spouse was interviewed at the same time. After completing the questionnaire and measurement, an 8 ml fasting blood sample was taken by a trained caregiver from respondents who had fasted for at least 8 h. A complete blood count (CBC) test is performed within 1–2 h of sampling. Whole blood samples were stored at 4°C for the subsequent determination of HbA1C. The remaining blood samples were separated into plasma and red blood cells and stored at −20°C. All the blood samples have been transported to Beijing and stored at −70°C at the Chinese Center for Disease Control for testing (29). The CHARLS began in 2011 with a cohort of 20,103 participants between the ages of 45 and 101 (Waves1). We excluded individuals who met any of the criteria at baseline (1) depressive symptom data missing, (2) one of the 13 indices missing, (3) age/sex/educational levels/marital status/current smoking/alcohol drinking/exercise/chronic diseases/live place/activities missing. Finally, 8,942 people aged 45 and older were included in our study. Figure 1 shows a flow diagram of the study individuals. All data are openly published as microdata at^1^ with no direct contact with all participants. Approval for this study was given by the medical ethics committee of Wannan Medical College (approval number 2021–3).

### Participants

Study subjects for this investigation were chosen from the CHARLS, Wave 1 (2011). The average age of the 8,942 participants participating in CHARLS was 59.07 years (standard deviation SD = 9.54, range 45–98 years). Males had a mean age of 60.16 years (SD = 9.26; range 45–98 years), while females had a mean age of 58.13 years (SD = 9.69; range 45–91 years).

## Baseline characteristics

### Covariates

Baseline characteristics including age, sex (1 = male; 2 = female), education (1 = illiterate; 2 = less than elementary school; 3 = high school; 4 = above vocational school), marital status (1 = married; 2 = single), living place (1 = rural; 2 = urban), smoking status (1 = no; 2 = former smoke; 3 = current smoke), drinking status (1 = no; 2 = less than once a month; 3 = more than once a month), taking activities (1 = no; 2 = yes), and having regular exercises (1 = no; 2 = less than exercises; 3 = regular exercises),and the counts of chronic diseases (0 = 0; 1 = 1–2; 2 = 3–14) were collected by self-report. The majority of factors were based on our earlier research investigations (30–35). “Having regular exercises” was defined as exercising at least 3 days a week and over 30 min a day, including moderate to intense physical activity and walking (34).

### Depressive symptom

The Center for Epidemiological Research Depression Scale (CES-D) in Chinese has been a good level of validity and reliability and is frequently used to assess depressive symptom in Chinese adults (36–39). The CES-D-10 has 10 items with 4 response options: (1) rarely or never (1 day); (2) sometimes or rarely (1–2 days); (3) occasionally or occasionally but not frequently (3–4 days); and (4) most or always (>1 day; 5–7 days). The values of the four options range from 0 to 3. A lower number denotes a lesser level of depressive symptom, and the total score goes from 0 to 30. A cutoff score ≥ 10 was used to identify respondents with significant depressive symptom (27, 40). The Cronbach alpha coefficient was 0.80 and the construct validity was 0.61 (31).

### Measurements

BMI was calculated based on the participants’ measured weight and height. BMI was divided into three categories under the Chinese standard definition: obesity (BMI ≥ 28 kg/m^2^), overweight (24 ≤ BMI <28 kg/m^2^), and underweight and normal (BMI < 24 kg/m^2^) (41). Waist circumference was measured with a flexible measuring tape at a level midway between the lower rib margin (35). The measurement method of conicity index was completed by waist circumference, weight, and height (42). BRI, ABSI, and VAI were calculating the following equations, it was important to note that VAI, unlike other anthropometrics, was divided into men and women (24, 43, 44). WHtR was defined as the waist circumference (m) divided by the height (m) (45). LAP was calculated slightly differently by subtracting a number (males: 65 cm, females: 58 cm) from waist circumference and multiplying by TG (46). CVAI was based on VAI to develop a more appropriate measure for Chinese people (47). The trained investigators measured the participants’ physical characteristics using standardized equipment, including their standing height, weight, and waist circumference [Index: height; Equipment: SecaTM213 Stadiometer Manufacturer/source: China Seca (Hangzhou) Co., Ltd. Index: weight; Equipment: OmronTMHN-286Scale Manufacturer/source: Krill Technology (Yangzhou) Co., Ltd. Index: waist circumference; Equipment: Soft Tape Measure Manufacturer/source: None. Venous blood sample: Standard blood collection materials] (29). The other anthropometric indexes were measured by the following formulas:

(1) BMI = $\frac{\mathrm{Weight}}{{\mathrm{Height}}^{2}}$

(2) WHtR = $\frac{\mathrm{Waist circumference}}{\mathrm{Height}}$

(3) Males: VAI = $\frac{\mathrm{Waist circumference}}{39.68+\left(1.88\times BMI\right)}\times \frac{TG}{1.03}\times \frac{1.31}{HDL}$

Females: VAI = $\;\frac{\mathrm{Waist circumference}}{36.58+\left(1.89\times BMI\right)}\times \frac{TG}{0.81}\times \frac{1.52}{HDL}$

(4) ABSI = $\frac{\mathrm{waist circumference}}{H{\mathrm{eight}}^{\frac{1}{2}}\times BMI^{\frac{2}{3}}}$

(5) BRI = $364.2-365.5\sqrt{1-\left(\frac{\mathrm{Waist circumference}÷{\left(2\pi \right)}^{2}}{{\left(0.5\times Height\right)}^{2}}\right)}$

(6) Males: LAP = [waist circumference (cm)-65] × TG (mmol/l)

Females: LAP = [waist circumference (cm)-58] × TG (mmol/l)

(7) Conicity Index = $\frac{\mathrm{Waist circumference}\left(m\right)}{0.019\sqrt{\frac{\mathrm{weight}\left(\mathrm{kg}\right)}{\mathrm{height}\left(m\right)}}}$

(8) Males:

CVAI = −267.93 + 0.68 × age+0.03 × BMI (kg/m^2^) + 4.00 × waist circumference (cm) + 22.00 × Log_10_TG (mmol/l) − 16.32 × HDL-C (mmol/l)

Females:

CVAI = −187.32 + 1.71 × age+4.32 × BMI (kg/m^2^) + 1.12 × waist circumference (cm) + 39.76 × Log_10_TG (mmol/l) − 11.66 × HDL-C (mmol/l)

(9) TyG index = Ln[(TG (mg/dl) × glucose (mg/dl)/2)]

(10) TyG-BMI = TyG × BMI

(11) TyG-waist circumference = TyG × Waist circumference

(12) TyG-WHtR = TyG × WHtR

### Statistical analysis

All the statistical analyses were analyzed using the SPSS software, version 25.0 (IBM SPSS, Armonk, NY, United States). Statistical significance was set at *p <* 0.05. To compare dichotomous or categorical variables, a chi-squared test was performed. Results of gender-stratified studies were also provided, as the connection between depressive symptom and obesity status differs in sexes. Continuous variables were measured using mean values and SD (continuous data). In order to evaluate the variations in mean distributions by sex, independent samples t-tests were utilized. After adjusting for age, education, marital status, current residence, current smoking, alcohol consumption, activity participation, regular exercise and chronic diseases, 13 indicators were used as independent variables and depressive symptom as dependent variables to evaluate the relationship between cardiometabolic indicators and depressive symptom. We found that there was no difference between these missing data and all data in gender, age and other indicators, so we adopted the direct deletion method for missing data. The unadjusted and adjusted relationships between anthropometrics and depressive symptom were evaluated using binary logistic regression, and odds ratios (ORs) and 95% confidence intervals (95% CI) were calculated after controlling for confounding factors. The ability of these indicators to identify depressive symptom was tested by drawing receiver operating curve (ROC) and calculating area under curve (AUC). The sensitivity, specificity, positive predictive value, negative predictive value, positive likelihood ratio, and negative likelihood ratio can all be calculated using the ROC curve. The cutoff points were chosen using the Youden index (sensitivity + specificity 1) to assess classification accuracy.

## Results

Table 1 shows the characteristics of participants with full samples. A total of 8,942 subjects were included in this study, of whom 4,146 (46.37%) were male and 4,796 (53.63%) were female. Most of them were 45–64 years old, there were significant differences between men and women in age, education, marital status, current smoking, alcohol drinking, number of chronic diseases, waist circumference, BMI, WHtR, VAI, ABSI, BRI, LAP, conicity index, CVAI, TyG index, TyG-BMI, TyG-waist circumference and TyG-WHtR (*p* < 0.05). However, the distribution of current residence, taking activities, and having regular exercises were not statistically significant between the male and female subgroups (*p* > 0.05). Because of these significant differences between males and females (*p* < 0.05), we performed the main analyses separately by sex.

**Table 1:** Characteristics of participants with full samples (*N* = 8,942).

Variables	Male	Female	Total	*t*/*χ*^2^	*p*
	*N* (%)	*N* (%)	*N* (%)		
*N*	4,146 (46.37)	4,796 (53.63)	8,942 (100.00)		
Age (years)					
45–54	1,225 (29.55)	1847 (38.51)	3,072 (34.35)	88.673	0.000
55–64	1,664 (40.14)	1793 (37.39)	3,457 (38.66)		
65–74	929 (22.41)	840 (17.51)	1769 (19.78)		
≥75	328 (7.91)	316 (6.59)	644 (7.20)		
Education					
Illiterate	540 (13.02)	1970 (41.08)	2,510 (28.07)	886.787	0.000
Less than elementary school	3,060 (73.81)	2,484 (51.79)	5,544 (62.00)		
High school	348 (8.39)	250 (5.21)	598 (6.69)		
Above vocational school	198 (4.78)	92 (1.92)	290 (3.24)		
Marital status					
Single	372 (8.97)	687 (14.32)	1,059 (11.84)	61.007	0.000
Married	3,774 (91.03)	4,109 (85.68)	7,883 (88.16)		
Current residence					
Rural	3,815 (92.02)	4,430 (92.37)	8,245 (92.21)	0.384	0.536
Urban	331 (7.98)	366 (7.63)	697 (7.79)		
Current smoking					
No	1,020 (24.60)	4,424 (92.24)	5,444 (60.88)	4272.142	0.000
Former smoke	705 (17.00)	89 (1.86)	794 (8.88)		
Current smoke	2,421 (58.39)	283 (5.90)	2,704 (30.24)		
Alcohol drinking					
No	1,831 (44.16)	4,227 (88.14)	6,058 (67.75)	2046.558	0.000
Less than once a month	454 (10.95)	238 (4.96)	692 (7.74)		
More than once a month	1861 (44.89)	331 (6.90)	2,192 (24.51)		
Taking activities					
No	2,004 (48.34)	2,362 (49.25)	4,366 (48.83)	0.743	0.389
Yes	2,142 (51.66)	2,434 (50.75)	4,576 (51.17)		
Having regular exercises					
No exercise	2,563 (61.82)	2,910 (60.68)	5,473 (61.21)	1.294	0.524
Less than exercises	786 (18.96)	945 (19.70)	1731 (19.36)		
Regular exercises	797 (19.22)	941 (19.62)	1738 (19.44)		
Chronic diseases (counts)					
0	1,372 (33.09)	1,394 (29.07)	2,766 (30.93)	22.141	0.000
1–2	2059 (49.66)	2,436 (50.79)	4,495 (50.27)		
3–14	715 (17.25)	966 (20.14)	1,681 (18.80)		
Waist Circumference	85.01 ± 9.78	85.71 ± 10.16	85.38 ± 9.99	−3.308	0.001
BMI	22.98 ± 3.60	24.04 ± 4.07	23.55 ± 3.89	−13.050	0.000
WHtR	0.52 ± 0.06	0.56 ± 0.07	0.54 ± 0.07	−32.232	0.000
VAI	3.96 ± 4.38	6.10 ± 5.76	5.11 ± 5.27	−19.914	0.000
ABSI	0.08 ± 0.01	0.08 ± 0.01	0.08 ± 0.01	−9.860	0.000
BRI	3.78 ± 1.14	4.66 ± 1.46	4.25 ± 1.39	−32.113	0.000
LAP	30.93 ± 33.27	44.01 ± 35.46	37.95 ± 35.07	−17.982	0.000
Conicity index	1.27 ± 0.08	1.30 ± 0.10	1.29 ± 0.09	−14.675	0.000
CVAI	96.09 ± 47.43	107.03 ± 43.55	101.96 ± 45.71	−11.296	0.000
TyG index	8.62 ± 0.66	8.72 ± 0.63	8.68 ± 0.65	−7.388	0.000
TyG-BMI	198.90 ± 39.18	210.31 ± 41.90	205.02 ± 41.05	−13.291	0.000
TyG-waist circumference	735.13 ± 117.70	749.36 ± 116.25	742.76 ± 117.13	−5.741	0.000
TyG-WHtR	4.48 ± 0.69	4.90 ± 0.76	4.71 ± 0.76	−27.338	0.000

BMI, body mass index; WHtR, waist to height ratio; VAI, visceral adiposity index; ABSI, a body shape index; BRI, body roundness index; LAP, lipid accumulation product; CVAI, cardio-ankle vascular index; TyG, triglyceride and glucose index; TyG-BMI, TyG related to BMI; TyG-waist circumference, TyG related to waist circumference; TyG-WHtR, TyG related to WHtR.

Table 2 shows the baseline characteristics of the study participants with and without depressive symptom by sex. According to the research results, the proportion of women suffering from depressive symptom was much higher (55.05%, compared to 41.12% for men). Men with depressive symptom had significant differences in age, education, marital status, current residence, alcohol drinking, taking activities, chronic diseases, waist circumference, BMI, WHtR, BRI, LAP, CVAI, TyG-BMI, TyG-waist circumference and TyG-WHtR (*p* < 0.05); women had significant differences in age, education, marital status, current residence, smoking, having regular exercises, chronic diseases, BMI, VAI, ABSI, LAP, conicity index, TyG-BMI, and TyG-waist circumference, (*p* < 0.05). There was no significant difference in the TyG index between subgroups of patients with and without depressive symptom, whether in men or women (*p* > 0.05).

**Table 2:** Baseline characteristics of the study participants with and without depressive symptom by sex.

Variables	Male (*N* = 4,146)		*χ* ^2^	*p*	Female (*N* = 4,796)		*χ* ^2^	*p*
*N* (%)	With depressive symptom *N* (%)	Without depressive symptom *N* (%)			With depressive symptom *N* (%)	Without depressive symptom *N* (%)		
*N*	1,705 (41.12)	2,441 (58.88)			2,640 (55.05)	2,156 (44.95)		
Age (years)								
45–54	476 (27.92)	749 (30.68)	8.220	0.042	966 (36.59)	881 (40.86)	11.665	0.009
55–64	676 (39.65)	988 (40.48)			1,015 (38.45)	778 (36.09)		
65–74	417 (24.46)	512 (20.98)			467 (17.69)	373 (17.30)		
≥75	136 (7.98)	192 (7.87)			192 (7.27)	124 (5.75)		
Education								
Illiterate	248 (14.55)	292 (11.96)	39.190	0.000	1,139 (43.14)	831 (38.54)	27.560	0.000
Less than elementary school	1,297 (76.07)	1,763 (72.22)			1,355 (51.33)	1,129 (52.37)		
High school	106 (6.22)	242 (9.91)			108 (4.09)	142 (6.59)		
Above vocational school	54 (3.17)	144 (5.90)			38 (1.44)	54 (2.50)		
Marital status								
Single	195 (11.44)	177 (7.25)	21.535	0.000	423 (16.02)	264 (12.24)	13.801	0.000
Married	1,510 (88.56)	2,264 (92.75)			2,217 (83.98)	1,892 (87.76)		
Current residence								
Rural	1,598 (93.72)	2,217 (90.82)	11.499	0.001	2,480 (93.94)	1,950 (90.45)	20.555	0.000
Urban	107 (6.28)	224 (9.18)			160 (6.06)	206 (9.55)		
Current smoking								
No	400 (23.46)	620 (25.40)	2.075	0.354	2,408 (91.21)	2016 (93.51)	9.461	0.009
Former smoke	292 (17.13)	413 (16.92)			59 (2.23)	30 (1.39)		
Current smoke	1,013 (59.41)	1,408 (57.68)			173 (6.55)	110 (5.10)		
Alcohol drinking								
No	795 (46.63)	1,036 (42.44)	7.617	0.022	2,312 (87.58)	1,915 (88.82)	2.436	0.296
Less than once a month	185 (10.85)	269 (11.02)			142 (5.38)	96 (4.45)		
More than once a month	725 (42.52)	1,136 (46.54)			186 (7.05)	145 (6.73)		
Taking activities								
No	876 (51.38)	1,128 (46.21)	10.735	0.001	1,330 (50.38)	1,032 (47.87)	2.997	0.083
Yes	829 (48.62)	1,313 (53.79)			1,310 (49.62)	1,124 (52.13)		
Having regular exercises								
No exercise	1,077 (63.17)	1,486 (60.88)	2.232	0.328	1,599 (60.57)	1,311 (60.81)	9.587	0.008
Less than exercises	312 (18.30)	474 (19.42)			555 (21.02)	390 (18.09)		
Regular exercises	316 (18.53)	481 (19.71)			486 (18.41)	455 (21.10)		
Chronic diseases(counts)								
0	415 (24.34)	957 (39.21)	158.415	0.000	613 (23.22)	781 (36.22)	144.079	0.000
1–2	871 (51.09)	1,188 (48.67)			1,362 (51.59)	1,074 (49.81)		
3–14	419 (24.57)	296 (12.13)			665 (25.19)	301 (13.96)		
Waist circumference	83.91 ± 9.77	85.78 ± 9.72	6.082	0.000	85.45 ± 10.24	86.02 ± 10.05	1.939	0.053
BMI	22.58 ± 3.59	23.27 ± 3.58	6.071	0.000	23.86 ± 4.05	24.27 ± 4.08	3.448	0.001
WHtR	0.51 ± 0.06	0.52 ± 0.06	4.541	0.000	0.56 ± 0.07	0.56 ± 0.07	0.610	0.542
VAI	3.89 ± 4.60	4.01 ± 4.22	0.876	0.381	5.95 ± 5.46	6.29 ± 6.10	2.015	0.044
ABSI	0.08 ± 0.01	0.08 ± 0.01	−0.724	0.469	0.08 ± 0.01	0.08 ± 0.01	−3.273	0.001
BRI	3.69 ± 1.13	3.84 ± 1.13	4.380	0.000	4.65 ± 1.44	4.68 ± 1.48	0.623	0.533
LAP	28.97 ± 33.44	32.30 ± 33.09	3.170	0.002	42.9 ± 34.15	45.37 ± 36.97	2.384	0.017
Conicity index	1.27 ± 0.08	1.27 ± 0.08	1.781	0.075	1.30 ± 0.10	1.30 ± 0.10	−1.973	0.048
CVAI	91.47 ± 47.07	99.32 ± 47.42	5.257	0.000	106.19 ± 42.77	108.05 ± 44.48	1.473	0.141
TyG index	8.61 ± 0.66	8.63 ± 0.66	0.935	0.350	8.71 ± 0.62	8.74 ± 0.64	1.847	0.065
TyG-BMI	195.12 ± 38.97	201.54 ± 39.11	5.212	0.000	208.31 ± 41.43	212.75 ± 42.35	3.649	0.000
TyG-waist circumference	724.47 ± 116.27	742.57 ± 118.14	4.886	0.000	745.65 ± 115.15	753.90 ± 117.45	2.445	0.015
TyG-WHtR	4.44 ± 0.68	4.52 ± 0.69	3.772	0.000	4.89 ± 0.75	4.92 ± 0.77	1.424	0.155

BMI, body mass index; WHtR, waist to height ratio; VAI, visceral adiposity index; ABSI, a body shape index; BRI, body roundness index; LAP, lipid accumulation product; CVAI, cardio-ankle vascular index; TyG, triglyceride and glucose index; TyG-BMI, TyG related to BMI; TyG-waist circumference, TyG related to waist circumference; TyG-WHtR, TyG related to WHtR.

Table 3 shows the associations of cardiometabolic indicators with depressive symptom. After controlling for age, education, marital status, current residence, current smoking, alcohol drinking, taking activities, having regular exercises, and chronic diseases, the odds ratio in waist circumference (OR = 0.978, 95%CI: 0.972–0.985), BMI (OR = 0.939, 95%CI: 0.920, 0.957), WHtR (OR = 0.042, 95%CI: 0.013–0.133), BRI (OR = 0.854, 95%CI: 0.805–0.905), LAP (OR = 0.996, 95%CI: 0.994–0.998), conicity index (OR = 0.369, 95%CI: 0.168–0.809), CVAI (OR = 0.996, 95%CI: 0.994–0.997), TyG-BMI (OR = 0.995, 95%CI: 0.993–0.997), TyG-waist circumference (OR = 0.998, 95%CI: 0.998–0.999), and TyG-WHtR (OR = 0.797, 95%CI: 0.724–0.877) in males and waist circumference (OR = 0.990, 95%CI: 0.984–0.995), BMI (OR = 0.968, 95%CI: 0.953, 0.982), WHtR (OR = 0.273, 95%CI: 0.111–0.672), VAI (OR = 0.985, 95%CI: 0.975–0.995), BRI (OR = 0.941, 95%CI: 0.903–0.980), LAP (OR = 0.997, 95%CI: 0.995–0.998), CVAI (OR = 0.997, 95%CI: 0.995–0.998), TyG index (OR = 0.856, 95%CI: 0.779–0.940), TyG-BMI (OR = 0.996, 95%CI: 0.995–0.998), TyG-waist circumference (OR = 0.999, 95%CI: 0.998–0.999), and TyG-WHtR (OR = 0.855, 95%CI: 0.789–0.926) in females were significantly correlated with depressive symptom. In addition, ABSI was not significantly associated with depressive symptom in either men or women.

**Table 3:** Associations of cardiometabolic indicators with depressive symptom.

Depressive symptom	Waist circumference	BMI	WHtR	VAI	ABSI	BRI	LAP	Conicity Index	CVAI	TyG index	TyG-BMI	TyG-waist circumference	TyG-WHtR
Male
Unadjusted OR (95% CI)	0.980 (0.974, 0.987)^**^	0.946 (0.929, 0.963)^**^	0.079 (0.026, 0.238)^**^	0.994 (0.980, 1.008)	1.045 (0.929, 1.175)	0.884 (0.837, 0.934)^**^	0.997 (0.995, 0.999)^*^	0.503 (0.237, 1.066)	0.996 (0.995, 0.998)^**^	0.956 (0.870, 1.051)	0.996 (0.994, 0.997)^**^	0.999 (0.998, 0.999)^**^	0.840 (0.768, 0.920)^**^
Adjusted OR (95% CI)	0.978 (0.972, 0.985)^**^	0.939 (0.920, 0.957)^**^	0.042 (0.013, 0.133)^**^	0.991 (0.976, 1.006)	1.002 (0.885, 1.134)	0.854 (0.805, 0.905) ^**^	0.996 (0.994, 0.998)^**^	0.369 (0.168, 0.809)^*^	0.996 (0.994, 0.997) ^**^	0.932 (0.845, 1.029)	0.995 (0.993, 0.997)^**^	0.998 (0.998, 0.999)^**^	0.797 (0.724, 0.877)^**^
Female
Unadjusted OR (95% CI)	0.994 (0.989, 1.000)	0.976 (0.962, 0.989)^**^	0.767 (0.328, 1.795)	0.990 (0.980, 1.000)^*^	1.163 (1.062, 1.274)^*^	0.988 (0.950, 1.027)	0.998 (0.996, 1.000)^*^	1.815 (1.002, 3.287)^*^	0.999 (0.998, 1.000)	0.919 (0.840, 1.005)	0.997 (0.996, 0.999)^**^	0.999 (0.999, 1.000)^*^	0.947 (0.879, 1.021)
Adjusted OR (95% CI)	0.990 (0.984, 0.995)^**^	0.968 (0.953, 0.982)^**^	0.273 (0.111, 0.672)^*^	0.985 (0.975, 0.995)^*^	1.086 (0.980, 1.204)	0.941 (0.903, 0.980) ^*^	0.997 (0.995, 0.998)^**^	0.950 (0.493, 1.830)	0.997 (0.995, 0.998)^**^	0.856 (0.779, 0.940)^*^	0.996 (0.995, 0.998)^**^	0.999 (0.998, 0.999)^**^	0.855 (0.789, 0.926)^**^

Odds ratios were adjusted for age, educational levels, marital status, live place, current smoking, alcohol drinking, activities, exercises, chronic diseases. ^*^p < 0.05, ^**^p < 0.001. BMI, body mass index; WHtR, waist to height ratio; VAI, visceral adiposity index; ABSI, a body shape index; BRI, body roundness index; LAP, lipid accumulation product; CVAI, cardio-ankle vascular index; TyG, triglyceride and glucose index; TyG-BMI, TyG related to BMI; TyG-waist circumference, TyG related to waist circumference; TyG-WHtR, TyG related to WHtR.

**Figure 2:**
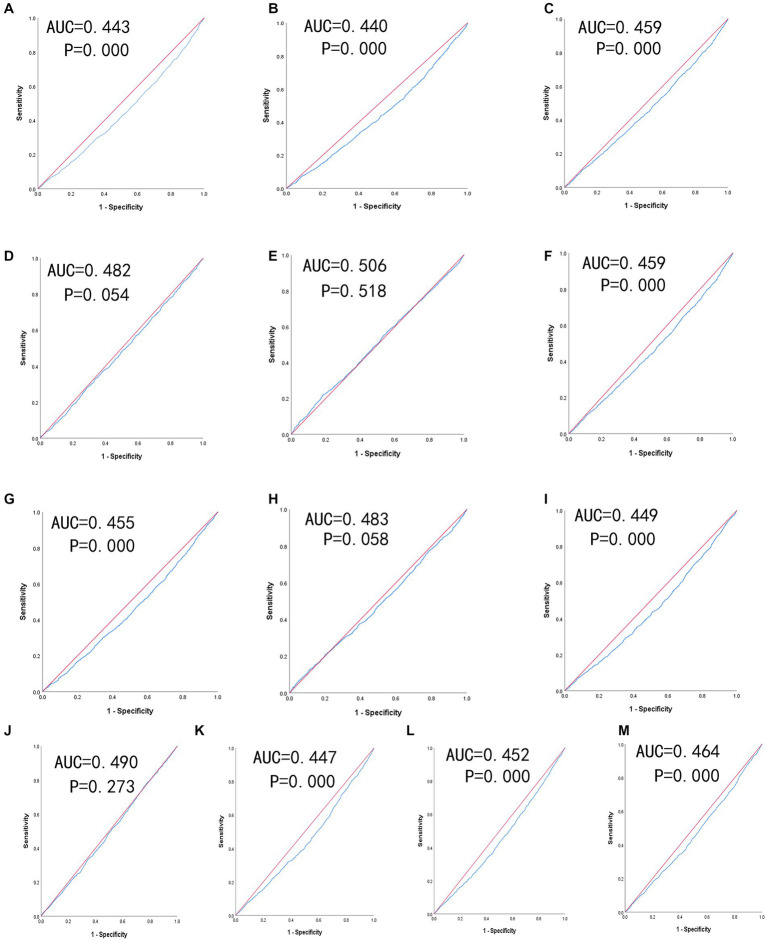
The ROC curves of each indicator in the prediction of depressive symptom risk in males. **(A)** waist circumference, **(B)** BMI, **(C)** WHtR, **(D)** VAI, **(E)** ABSI, **(F)** BRI, **(G)** LAP, **(H)** conicity index, **(I)** CVAI, **(J)** TyG-index, **(K)** TyG-BMI, **(L)** TyG-waist circumference, **(M)** TyG-WHtR.

**Figure 3:**
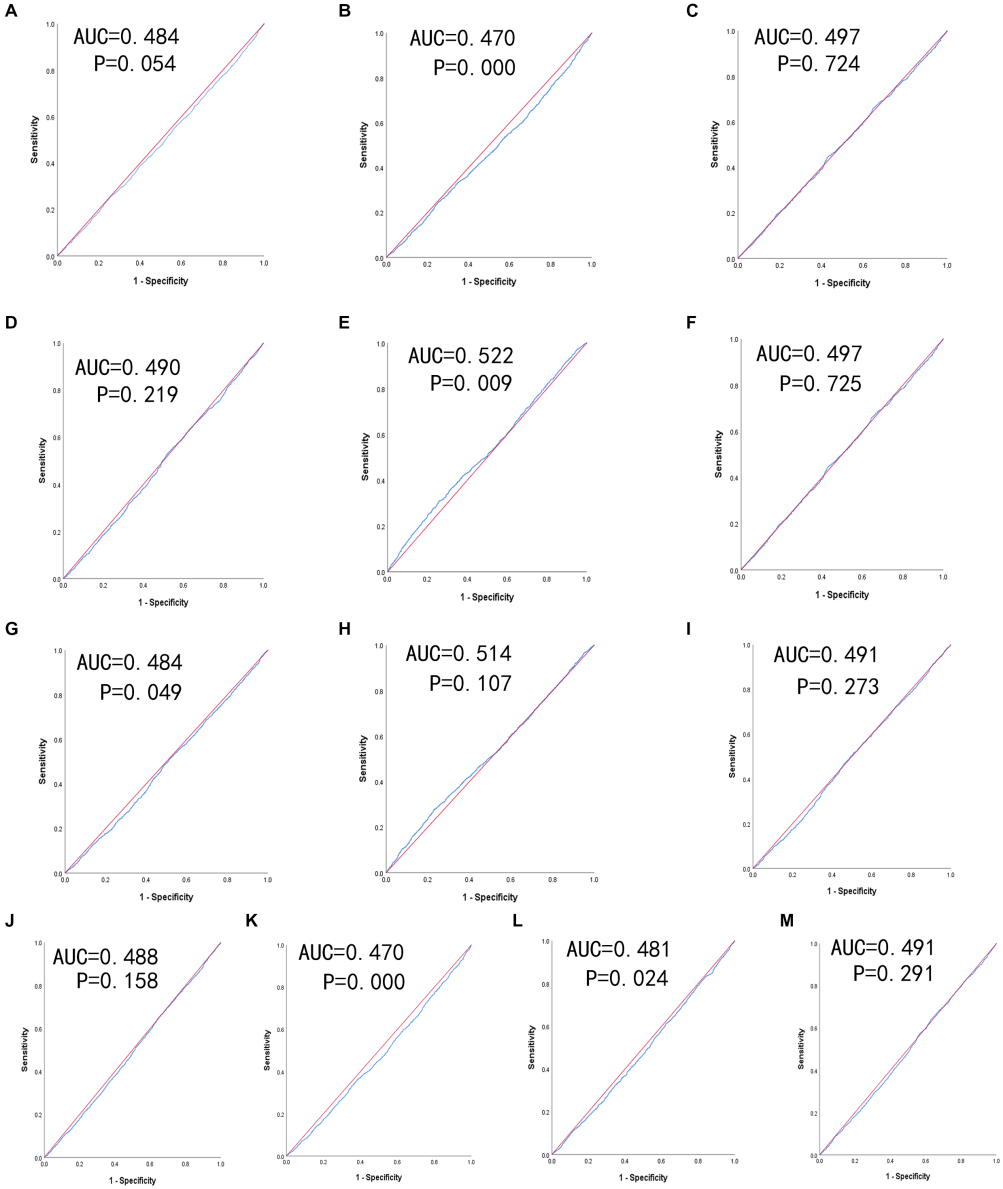
The ROC curves of each indicator in the prediction of depressive symptom risk in females. **(A)** waist circumference, **(B)** BMI, **(C)** WHtR, **(D)** VAI, **(E)** ABSI, **(F)** BRI, **(G)** LAP, **(H)** conicity index, **(I)** CVAI, **(J)** TyG-index, **(K)** TyG-BMI, **(L)** TyG-waist circumference, **(M)** TyG-WHtR.

Table 4 shows the predictive value is evaluated by the ROC curve and AUC. The ROC curves of each indicator in predicting depressive symptom risk in men and women are shown in Figures 2, 3 respectively. In men, the AUCs of waist circumference, BMI, WHtR, BRI, LAP, CVAI, TyG-BMI, TyG-waist circumference, and TyG-WHtR showed weak but significant power in predicting depressive symptom (*p* < 0.05). It can be observed from the table that WHtR (AUC = 0.459, SE = 0.009, 95% CI = 0.441–0.476, and optimal cut-off = 0.754) and BRI (AUC = 0.459, SE = 0.009, 95% CI = 0.441–0.476, and optimal cut-off =9.371) had similar predictive values. In contrast, the AUC of ABSI did not reach statistical significance (*p* = 0.518). In women, the AUCs of BMI, LAP, TyG-BMI, and TyG-waist circumference showed weak but significant power in predicting depressive symptom (*p* < 0.05). Similarly, the predictive values were similar for the BMI (AUC = 0.470, SE = 0.008, 95% CI = 0.453–0.486, and optimal cut-off = 33.997), TyG-BMI (AUC = 0.470, SE = 0.008, 95% CI = 0.454–0.487, and optimal cut-off =113.997). In addition, there was no statistical significance in the AUC of VAI, conicity index and TyG index between men and women (*p* > 0.05).

**Table 4:** Cut-off between area under curve, sensitivity, and specificity for cardiometabolic indicators to detect depressive symptom by sex.

*N* = 8,942	Waist circumference	BMI	WHtR	VAI	ABSI	BRI	LAP	Conicity Index	CVAI	TyG index	TyG-BMI	TyG-waist circumference	TyG-WHtR
Male
Area under curve	0.443	0.440	0.459	0.482	0.506	0.459	0.455	0.483	0.449	0.490	0.447	0.452	0.464
Std. error	0.009	0.009	0.009	0.009	0.009	0.009	0.009	0.009	0.009	0.009	0.009	0.009	0.009
95%CI	0.425,0.460	0.422,0.457	0.441,0.476	0.465,0.500	0.488,0.524	0.441,0.476	0.437,0.472	0.465,0.501	0.432,0.467	0.472,0.508	0.429,0.465	0.434,0.470	0.446,0.481
*p*-value	0.000	0.000	0.000	0.054	0.518	0.000	0.000	0.058	0.000	0.273	0.000	0.000	0.000
Optimal cutoffs	116.400	47.157	0.754	25.881	0.086	9.371	187.923	1.387	244.113	8.146	317.179	1170.087	6.710
J-Youden	0.001	0.001	0.001	0.006	0.036	0.001	0.003	0.014	0.001	0.008	0.001	0.002	0.003
Sensitivity (%)	0.20%	0.20%	0.10%	1.10%	21.80%	0.10%	0.90%	8.70%	0.20%	76.50%	0.80%	0.20%	0.40%
Specificity (%)	99.90%	99.90%	100%	99.50%	81.80%	100.00%	99.40%	92.70%	99.90%	24.30%	99.30%	100%	99.90%
(+) Likelihood ratio	2.000	2.000	0.000	2.200	1.198	0.000	1.500	1.192	2.000	1.011	1.143	0.000	4.000
(−) Likelihood ratio	0.999	0.999	0.999	0.994	0.956	0.999	0.997	0.985	0.999	0.999	0.999	0.998	0.997
Female
Area under curve	0.484	0.470	0.497	0.490	0.522	0.497	0.484	0.514	0.491	0.488	0.470	0.481	0.491
Std. error	0.008	0.008	0.008	0.008	0.008	0.008	0.008	0.008	0.008	0.008	0.008	0.008	0.008
95%CI	0.467,0.500	0.453,0.486	0.481,0.513	0.473,0.506	0.505,0.538	0.481,0.513	0.467,0.500	0.497,0.530	0.474,0.507	0.472,0.505	0.454,0.487	0.465,0.498	0.475,0.508
*P*-value	0.054	0.000	0.724	0.219	0.009	0.725	0.049	0.107	0.273	0.158	0.000	0.024	0.291
Optimal cutoffs	114.750	33.997	0.570	4.170	0.086	4.764	5.650	1.358	104.859	8.453	113.997	533.673	4.572
J-Youden	0.003	0.002	0.016	0.011	0.051	0.016	0.004	0.047	0.008	0.004	0.001	0.001	0.011
Sensitivity (%)	0.60%	1.30%	44.50%	52.80%	31.00%	44.50%	98.90%	27.60%	50.10%	64.70%	100.00%	98.70%	64.20%
Specificity (%)	99.70%	98.90%	57.10%	48.30%	74.10%	57.10%	1.50%	77.10%	50.70%	35.70%	0.10%	1.40%	36.90%
(+) Likelihood ratio	2.000	1.182	1.037	1.021	1.197	1.037	1.004	1.205	1.016	1.006	1.001	1.001	1.017
(−) Likelihood ratio	0.997	0.998	0.972	0.977	0.931	0.972	0.733	0.939	0.984	0.989	0.000	0.929	0.970

BMI, body mass index; WHtR, waist to height ratio; VAI, visceral adiposity index; ABSI, a body shape index; BRI, body roundness index; LAP, lipid accumulation product; CVAI, cardio-ankle vascular index; TyG, triglyceride and glucose index; TyG-BMI, TyG related to BMI; TyG-waist circumference, TyG related to waist circumference; TyG-WHtR, TyG related to WHtR.

## Discussion

With the aging problem becoming more and more serious, the influences of depressive symptom on the quality of life and survival of older adults cannot be ignored. Therefore, efficient and simple screening tools are essential to identify patients with depressive symptom. In our study, we found that there was a significant negative correlation between cardiometabolic indicators and depressive symptom, but there were differences in gender (male and female). For example, waist circumference (OR = 0.978, 95%CI: 0.972–0.985), WHtR (OR = 0.042, 95%CI: 0.013–0.133), BRI (OR = 0.854, 95%CI: 0.805–0.905) and CVAI (OR = 0.996, 95%CI: 0.994–0.997) were negatively correlated with depressive symptom in men, but not in women. In addition, BMI, TyG-waist circumference, TyG-BMI and LAP had a certain ability to predict depressive symptom.

Our data showed that BMI-adjusted OR in men and women was inversely associated with depressive symptom, with changes in BMI indicating a higher risk of depressive symptom, the results of this study were similar to several Asian studies (19, 48). In addition, studies have shown that inflammatory cytokines are associated with BMI Obesity and central fat can increase plasma concentrations of inflammatory cytokines leading to low levels of systemic inflammation (49–51), and an increase in inflammatory cytokines can make depressive symptom and anxiety worse (52). So, in our study, something like BMI could reduce depressive symptom by screening patients for depressive symptom, and Vittengl et al. (53) have shown the same idea.

In recent years, the TyG index has emerged as a novel marker for insulin resistance. Many observational studies (54–56) found that diabetes and insulin resistance can exacerbate depressive symptom. In South Korea, a sizable cross-sectional investigation found that depressive symptom risk increased by 4% in young people and 17% in non-diabetics when insulin resistance was raised (57). Multiple studies (58, 59) have found that high levels of Tyg-related indicators perform better in assessing depressive symptom in adults. In our study, TyG-related factors such as TyG-BMI, and TyG-waist circumference can provide a broader basis for cardiometabolic indicators to estimate depressive symptom. Many earlier studies (60–62) shared similar views to our findings, but they involved fewer participants. With the increasing prevalence of chronic diseases in older adults, the correlation between the TyG index and depressive symptom can be further explored in future studies.

Among other measures, LAP was also significant in predicting depressive symptom. LAP was first proposed by Kahn et al. (46). With the increase of abdominal fat accumulation, TG concentration increased, and the association of metabolic insulin resistance was enhanced (63, 64) and LAP was used as a predictor of insulin resistance (65, 66). In this study, LAP was negatively correlated with depressive symptom in males (OR = 0.996, 95%CI: 0.994–0.998), as well as in females (OR = 0.997, 95%CI: 0.995–0.998). Moreover, in ROC analysis, the predicted values were statistically significant (*p* < 0.05), which could be used as a valuable predictor of depressive symptom.

In addition, the study showed that ABSI was not an ideal predictor compared to BMI and LAP. According to Lotfi et al. (67), there was a substantial positive correlation between ABSI and psychological discomfort in women (OR = 1.40; 95%CI: 1.07, 1.84), but not present in men. On the other hand, they found no significant association between BRI and depressive symptom. These findings were inconsistent with our results. In our study, there was no correlation between ABSI and depressive symptom, which was consistent with Hadi et al. (68). In addition, by observing the ROC curve of cardiometabolic indicators, it is found that the area of the ROC curve of male ABSI had no statistical significance (*p* > 0.05). In contrast, one study (69) showed superior predictive power and a significant association between BRI and high cardiometabolic risk, and BRI was inversely associated with the incidence of depressive symptom in our study.

Interestingly, there was a significant correlation between waist circumference, WHtR, and depressive symptom in our study. Consistent with our study, Liao et al. (70) found that the dose–response relationship between waist circumference and depressive symptom showed a linear trend. Similarly, among overweight and obese Americans, those with larger waists or bellies were more likely to have severe depressive symptom (71). In prospective research with 6,355 people, WHtR was assessed as the best predictor of cardiovascular risk and mortality (followed by waist circumference and WHR) (72). In Zhi et al.’s (73) study, abdominal obesity was only associated with depression in adult Iranian women. On the contrary, in our study, we found that waist circumference and WHtR had some significance in predicting depressive symptom in men. Waist circumference and WHtR measurements were simple and low-cost, allowing medical professionals to use them as reference factors for future depressive symptom in men.

VAI is the visceral cardiometabolic index, studies (74) showed that VAI reflected the deposition degree of adipose tissues, it used to be common in Caucasians, but VAI had a poor correlation with adipose tissue area in Chinese (75). A cross-sectional study of adults in the United States found that for every unit increase in VAI, the incidence of clinical depression increased by 14% (25). However, in our study, VAI was weakly associated with depressive symptom and could not be used as a predictor of depressive symptom in both men and women. In 2016, Xia et al. (47) developed a clinical indicator, CVAI, to predict the visceral fat region in Chinese adults, which can be used to assess metabolic health in Asians. This was similar to our study, in which CVAI was negatively correlated with depressive symptom. In addition, our data showed that CVAI can be used as a predictor of depressive symptom in men, but not in women.

By contrast, conicity index was not an ideal predictor. Longitudinal studies have confirmed that the severity of depressive symptom correlates with the risk of Alzheimer’s disease, and that the risk of major depressive symptom is higher (76, 77). This was consistent with our findings that conicity index was not associated with depressive symptom. Interestingly, Confortin et al. (78) reported that in addition to conicity index, BMI, waist circumference and WHtR had up to one-third independent association with dementia. Meanwhile, in the older adults (aged 61–90) of the Federal District (Brazil), Brito et al. discovered a correlation between dementia and WHtR but no correlation with conicity index (79).

This study mainly discussed the correlation and prediction between cardiometabolic indicators and depressive symptom, deepened the importance of early detection of depressive symptom based on previous studies, made up for the deficiency of single content of previous studies to a certain extent, and enriched the epidemiological prevention and treatment of depressive symptom. In the field of public health, our findings can provide a reference for clinical work, public health consultation, and identification of high-risk groups for preventive treatment. Therefore, clinicians should monitor depressive symptom in obese populations. Such monitoring can lead to the prevention or early diagnosis of depressive symptom and can subsequently reduce the high disease burden in the population.

### Strengths and limitations of the study

Our study has several advantages. In the past, a single index was used to predict the prevalence of depressive symptom, which may cause bias. In this study, we compared the relation of 13 cardiometabolic indicators on depressive symptom, which was helpful for early screening. Secondly, most of the indicators in this study were simple and easy to operate, which can be used in clinical large-scale. In addition, this study included 8,942 respondents aged 45 and above, which ensured the accuracy of this study and has great theoretical and practical significance for the prevention and control of depressive symptom. However, some limitations should be considered. Firstly, the cross-sectional design limits study of the causal relationship between cardiometabolic indicators and depressive symptom. Secondly, although several covariables were considered in our analysis, some residual confounding factors may still influence the results. Thirdly, the data in this study were collected in 2011 and may not reflect the updated relationships between variables; however, the data could contribute to estimating the trend change by adding to the representative evidence and provide a comparison for future intervention and policymaking.

## Conclusion

In summary, our findings suggested that most cardiometabolic indicators were associated with depressive symptom. BMI, LAP, TyG-BMI, and TyG-waist circumference predict the correlation of depressive symptom in our study. For example, we can prevent or delay the onset and development of depressive symptom by changing the BMI of middle-aged and older adults. The results of this study may have important implications for the prevention of depressive symptom. Our findings highlight the need to raise awareness of depressive symptom and improve healthcare. Especially in the case of the increasing prevalence of depressive symptom year by year, it can further prevent the cognitive dysfunction of older adults and improve the overall quality of life of the older adults. At the same time, it lays a solid foundation for further research on depressive symptom in the future.

## Data availability statement

The original contributions presented in the study are included in the article/supplementary material, further inquiries can be directed to the corresponding author/s.

## Ethics statement

The studies involving human participants were reviewed and approved by medical ethics committee of Wannan Medical College (approval number 2021–3). The patients/participants provided their written informed consent to participate in this study.

## Author contributions

LZ conceived and designed the research. YW wrote the paper. YW and LZ analyzed the data. YW, XZ, YLi, JG, YM, XY, LZ, HL, LG, JinlL, YLe, XL, LS, LY, TY, CW, DZ, JingL, ML, and YH revised the paper. All authors contributed to the article and approved the submitted version.

## Funding

CHARLS was supported by the NSFC (70910107022 and 71130002) and National Institute on Aging (R03-TW008358-01; R01-AG037031-03S1), and World Bank (7159234) and the Support Program for Outstanding Young Talents from the Universities and Colleges of Anhui Province for LZ (gxyqZD2021118).

## Conflict of interest

The authors declare that the research was conducted in the absence of any commercial or financial relationships that could be construed as a potential conflict of interest.

## Publisher’s note

All claims expressed in this article are solely those of the authors and do not necessarily represent those of their affiliated organizations, or those of the publisher, the editors and the reviewers. Any product that may be evaluated in this article, or claim that may be made by its manufacturer, is not guaranteed or endorsed by the publisher.

## Footnotes

^1^ http://charls.pku.edu.cn/CHARLS

## Abbreviations

BMI: body mass index
WHtR: waist-height ratio
VAI: visceral adiposity index
CVAI: cardio-ankle vascular index
LAP: lipid accumulation product
ABSI: a body shape index
BRI: body roundness index
TyG: triglyceride glucose
TyG-WHtR: triglyceride glucose-waist-height ratio
TyG-BMI: triglyceride glucose-body mass index
TyG-waist circumference: triglyceride glucose-waist circumference
CHARLS: China Health and Retirement Longitudinal Study
SD: standard deviation
CES-D: Center for Epidemiological Research Depression Scale
OR: odds ratio
95% CI: 95% confidence intervals
ROC: receiver operator curve
AUC: area under curve
TG: triglyceride
